# Computational Investigation of Ginsenoside F1 from *Panax ginseng* Meyer as p38 MAP Kinase Inhibitor: Molecular Docking and Dynamics Simulations, ADMET Analysis, and Drug Likeness Prediction

**Published:** 2018

**Authors:** Hae-Yong Noh, Jing Lu, Muhammad Hanif Siddiqi, Sathishkumar Natatajan, Sera Kang, Sungeun Ahn, Yeon-Ju Kim, Deok-Chun Yang

**Affiliations:** a *Department of Oriental Medicinal Biotechnology, College of Life Sciences, Kyung Hee University, Giheung-gu, Yongin-si, Gyeonggi-do, Republic of Korea. *; b *Graduate School of Biotechnology, College of Life Science, Kyung Hee University, Seocheon, Giheung-gu, Yongin-si, Gyeonggi-do, Republic of Korea.*; 1 J.L. and H.YN.contributed equally to this work.

**Keywords:** Panax ginseng, p38 MAP kinase inhibitor, G-F1, ADMET, Docking

## Abstract

Ginsenoside F1 (G-F1) is biologically an active compoud isolated from Korean *Panax ginseng* Meyer. In the present study, the potential therapeutic effect of G-F1 were investigated by computational target fishing approaches including ADMET prediction, biological activity prediction from chemical structure, molecular docking, and molecular dynamics methods. Results were suggested to express the biological activity of G-F1 against p38 MAP kinase protein. The p38 MAP kinase protein is an important signal transducing enzyme involved in many cellular regulations, including signaling pathways, pain and inflammation. Numerous studies are shown that an abnormal activation of p38 MAP kinase leads to variety of diseases. The pharmacokinetic result proves that G- F1 can act non-toxic drug like molecule. In addition, molecular level interaction results of G- F1 with p38 MAP kinase active (binding) sites residues clearly defines its inhibitory action on p38 MAP kinase. Further, molecular dynamics study also supported p38 MAP kinase and G-F1 structural stability. Findings from out study will assist to discover the active drug like molecules from *Panax ginseng* with help of molecular modeling techniques.

## Introduction

Parkinson’s disease (PD) is one of the major neurological disorder, reduces the dopaminergic neurons in substantia nigra of ventral midbrain due to the accumulation of insoluble aggregated alpha-synuclein (α-Syn) in brain stem, spinal cord, and cortex. In pathological condition of PD there is successive reduction of dopamine (DA) supply levels in the striatum that causes imbalance between neurotransmitters like acetylcholine and DA as well as degeneration of non-dopaminergic mechanism such as cholinergic, noradrenergic and serotonergic systems in PD person (1,2). Probably numerous motor and non-motor characteristics symptoms including rigidity, tremors, depression, dementia, and sleep abnormalities are the consequence of deterioration of both dopaminergic and non-dopaminergic mechanism (3). The pathogenesis and etiology of PD are not entirely understood yet. Even-though no model up to now has been able to elucidate all the pathological conditions of PD. However, the three main drug development advances, rotenone, 1-methyl-4-phenyl-1,2,3,6-tetrahydropyridine (MPTP) and 6-hydroxydopamine (6-OHDA) are the most vital therapeutic agents for the management of PD as *in-vitro* and *in-vivo* (4). Dopamine transporter (DAT) produces free radicals and complex-I inhibition by taken up 6-OHDA, 1-methyl-4-phenylpyridinium (MPP+) by the action of Monoamine oxidase B (MAOB) that can be accumulated by mitochondria (4). In recent decades, it has suggested that Mitogen-activated protein kinase (MAPK) pathways are the central inducers that transmit extracellular signals from the membrane to the nucleus in neurodegenerative disease and including PD and apoptosis. Different extracellular stimuli such as environmental stressors, cellular injury, and inflammatory cytokines are responsible for activation of serine/threonine protein, which causing different neuronal cell death including differentiation, proliferation and apoptosis (5, 6). It has been suggested that the two vital members of the MAPK signaling cascades such as c-Jun N-terminal kinases (JNK) and p38 mitogen-activated protein kinases (p38MAPK) mediate neurodegeneration in PD and Alzheimer’s disease (AD) (7, 8). Several studies have found that activation of p38, a pivotal member of the mitogen-activated protein kinase (MAPK) super family plays a central role in the pathogenesis of neurological disorders such as Parkinson’s disease, Alzheimer’s disease and inflammatory diseases (9). It has also been reported that the p38 activation causes the activation of inflammatory cytokines such TNFα, IL-1β, cyclooxygenase (COX)-2, IL-6, IL-12 and IFN-γ, which play important roles in autoimmune, neurodegenerative and cardiovascular diseases (10-12). 

Several *in-vivo* (Wistar rat, C57/BL6 mice,) and *in-vitro* (SH-SY5Y and PC12 cells) models might assist as valuable measurement tools for the evaluation and efficacy for development of novel therapeutic agents for treatments of PD. Widespread study of these *in-vitro* and *in-vivo* models have provided significant cellular agents of cell apoptosis comprising excitotoxicity, mitochondrial dysfunction, neuro-inflammation, nitric oxide and oxidative stress (13, 14). 

Various therapeutic agents are used for enhancements in dopaminergic remedies and the development of non-dopaminergic medications to treat PD, however, they are associated with side effects. In this regard, natural compounds are potent sources for treatment and management of PD. Currently the root of *Panax ginseng* Meyer (*P. ginseng*) is used to treat human disorder as an oriental medicine. *P. ginseng* has three types of saponins commonly referred to as ginsenosides: protopanaxadiol (PPD), protopanaxatriol (PPT), and oleanolic acid (15). Many studies have revealed pharmacological and biological activities of different ginsenosides such as anti-oxidant, anti-inflammatory, and anti-proliferative effects (16). It has been reported that compound K and ginsenoside Rg3 have anti-tumor and anti-cancer activity (17, 18). Recently, we have reported that ginsenoside Rh1 and Rg5:Rk1 have anti-osteoporotic activity by elevating osteoblasts differentiation and mineralization in MC3T3-E1 cells (19, 20). In addition, it has been reported that ginsenoside Rh2 inhibits metabolic disorders like obesity through the adenosine monophosphate-activated protein kinase (AMPK) signaling pathway (21). Ginsenoside F1 is a *P. ginseng* metabolite produced by enzymatic modification of ginsenoside Rg1, recently Tawab *et al*; reported that compound K, ginsenosides Rh1 and F1 were detected in the blood and urine of humans after oral administration of ginseng extract (22, 23). As several ginsenosides have been suggested for treatment of different diseases, however, there is no evidence published to support the efficacy of G-F1 in Parkinson’s disease. Thus, to investigate the role of G-F1 in Parkinson’s disease, we performed *in silico* docking of p38 MAPK with G-F1 and calculated their interaction properties including binding energy, hydrogen bonds and active site binding mode. We also investigated the molecular dynamics of protein-ligand complex in order to evaluate its binding stability. In addition, we predicted the absorption, distribution, metabolism, excretion, and toxicity (ADMET) of G-F1. The primary aims of this study were to determine the biological activities of G- F1 and its molecular interactions with p38 MAPK.

## Experimental part

*Materials and Methods*

*Preparation of Protein and ligand molecules*

**Figure 1:**
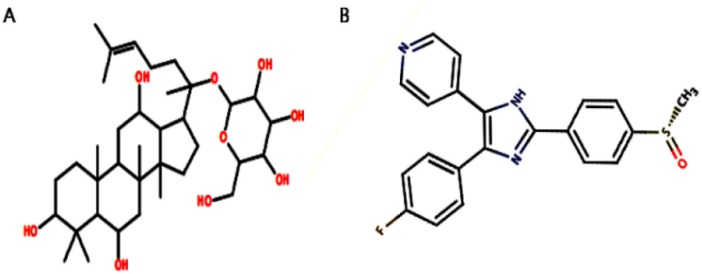
Two-dimensional structures of (A) G-F1, and (B) SB203580 compound

We retrieved the structure of G-F1 from our own in-house *P. ginseng* saponin database. Its two-dimensional (2D) structure was drawn using ACD/ChemSketch (http://www.acdlabs.com) and converted to a three-dimensional (3D) structure by the OpenBabel program (24). We also retrieved the p38 MAPK inhibitor SB203580 from the p38 MAPK crystal complex structure to use as control ligand for docking simulation. Figure 1. shows the 2D structures of G-F1 and SB203580 ligand. These two compounds were energy-minimized using the 200 steps of steepest descent (25) followed by the conjugate gradients method (26) using the universal force field (UFF) (27) as carried out by the PyRx program (28).

*ADMET screening for drug likeness*

Lipinski’s rule of five (29) was used to determine drug-likeness. This rule is based on the observation that most orally administered drugs have molecular weight less than 500, a distribution ratio less than 5, less than 5 hydrogen bond donors, and less than 10 hydrogen bond acceptors. The ADMET profile of G-F1 was determined studied using the Qikprop 3.0 module encoded by the Schrodinger program (http://www.schrodinger.com). This program generates both physicochemical and pharmacokinetically relevant properties based on the structure of G-F1. Qikprop 3.0 allows comparison of a particular molecule’s properties with those of 95% of known drugs. The hepatotoxicity and CYP2D6 inhibition scores were calculated using the ADMET module available from the DS 2.5 program (http://accelrys.com). The ADMET prediction steps were followed from our previous publications (30). 

**Figure 2:**
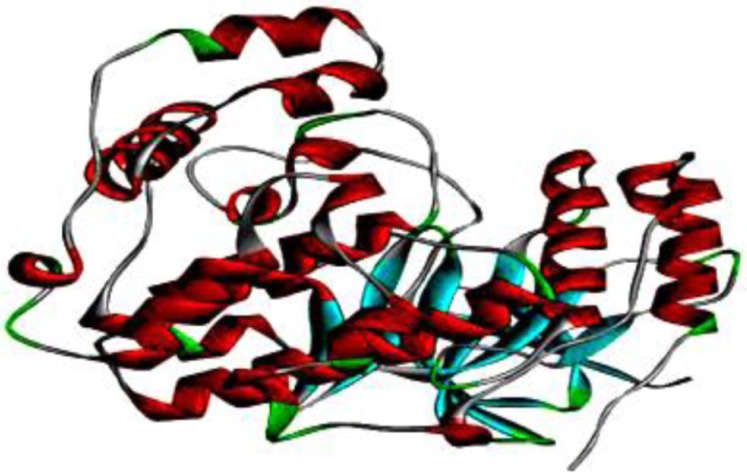
Three-dimensional crystal structure of p38 MAPK. This solid ribbon represents colors based on their secondary structure. Red represents helices, blue represents beta sheets, green represents turns, and white represents coils

**Figure 3:**
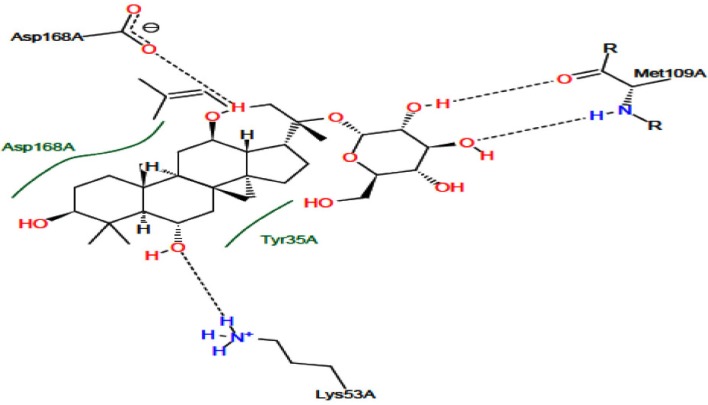
Docking interaction of p38 MAPK with G-F1. The total lines indicate hydrogen bonds between G-F1 and p38 MAPK

**Figure 4:**
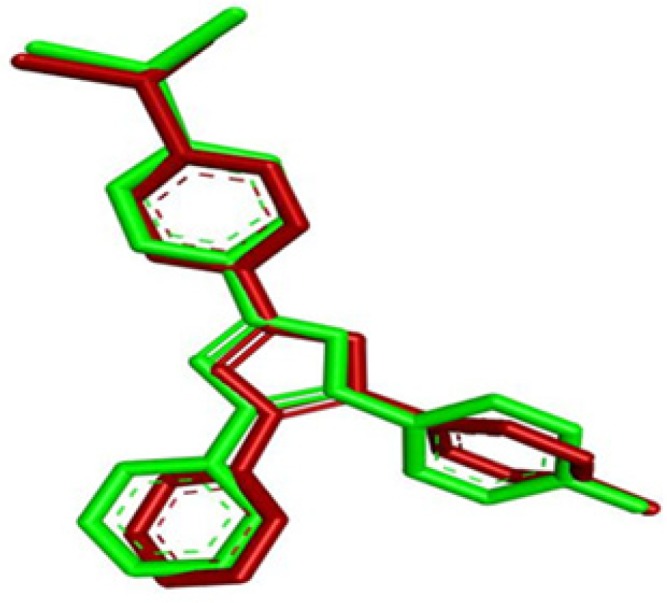
Superimposition results of SB203580 reproducibility. Green represents the experimental structure, red represents structure after docking

**Figure 5:**
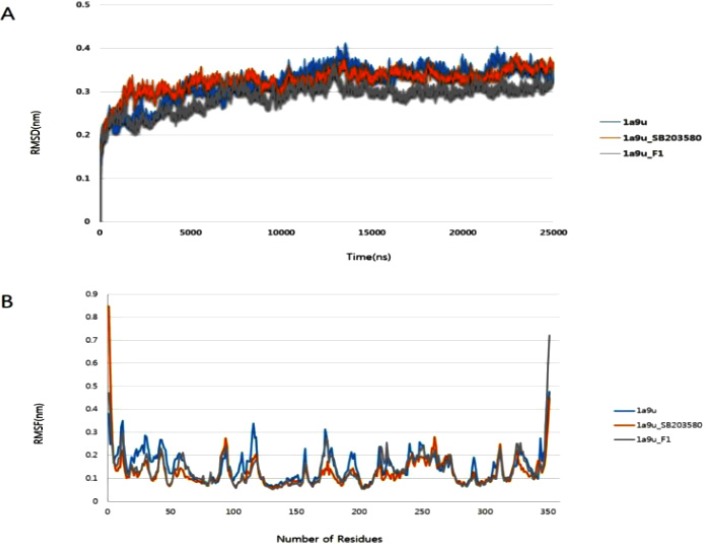
Molecular dynamics results analysis lasting 25 ns. (A) Root mean square deviation values of p38 MAPK enzyme and p38 MAPK complexes, (B) Root mean square fluctuation values of Cα atoms against residue numbers

**Table 1:** ADMET values of G-F1

**Principal descriptors**	**G-F1**	**Range of 95% of known drugs**
Log K has serum protein binding	0.063	-1.5/1.5
Log BB for brain/blood	-2.602	-3.0/1.2
HERG K+ channel blockage (log IC50)	-4.607	Concern below -5
Apparent caco-2 permeability	65.352	<25 poor, >500 great
Apparent MDCK permeability	25.929	<25 poor, >500 great
QP log Kp for skin permeability	-4.382	-8.0 to -1.0, Kp in cm/h
Human GI absorption (%)	48.05	<25% is poor
Lipinski rule of 5 violations	2	Max=4
Jorgensen rule of 3 violations	1	Max=3
Hepatotoxicity	0	0=non-toxic, 1=toxic
CYP2D6 probability	0.277	0=non-inhibitor, 1=inhibitor

**Table 2:** Predicted Biological activity of G-F1 including active (Pa) and inactive (Pi) probability scores

**No.**	**Pa**	**Pi**	**Activity**
1	0,995	0,001	Caspase 3 stimulant
2	0,992	0,001	Apoptosis antagonist
3	0,982	0,001	Chemopreventive
4	0,981	0,001	Antithrombotic
5	0,976	0,000	Dementia treatment
6	0,975	0,000	Vascular dementia treatment
7	0,964	0,000	CYP2C9 inducer
8	0,958	0,001	Anticarcinogenic
9	0,937	0,002	Hepatoprotectant
10	0,931	0,003	Alkenylglycerophosphocholine hydrolase inhibitor
11	0,927	0,004	Apoptosis agonist
12	0,917	0,003	Immunostimulant
13	0,915	0,002	CYP3A4 inducer
14	0,911	0,002	CYP3A inducer
15	0,900	0,003	Cholesterol antagonist
16	0,898	0,002	Antiulcerative
17	0,860	0,006	Antineoplastic
18	0,838	0,002	Transcription factor NF kappa B stimulant
19	0,838	0,002	Transcription factor stimulant
20	0,843	0,011	Benzoate-CoA ligase inhibitor
21	0,833	0,003	Antioxidant
22	0,832	0,010	Beta-adrenergic receptor kinase inhibitor
23	0,804	0,004	Immunosuppressant
25	0,800	0,003	Nitric oxide antagonist
26	0,787	0,008	Anti-inflammatory
27	0,292	0,073	Neurotrophic factor enhancer
28	0,509	0,017	Interleukin 2 agonist

**Table 3.** Docking energy and hydrogen bond interactions of active sites

**Compound**	**Docking energy (Kcal/mol)**	**No. of hydrogen bonds**	**Amino acid involved in interaction**	**H-bond distance (Å)**	**H-bond donor**	**H-bond acceptor**
SB203580	-8.02	2	Lys53	2.92	Lys53:HZ2	SB203580:NC3
			Met109	2.75	Met109:HN	SB203580:NB1
G-F1	-7.32	4	Lys53	1.98	Lys53:HZ3	F1:O
			Met109	1.89	Met109:HN	F1:O
				1.98	F1:H	Met109:O
			Asp168	2.10	F1:H	Asp168:OD2

*In silico prediction of biological activity*

We used the prediction of activity spectra for substances (PASS) online server (http://www.pharmaexpert.ru/passonline/) (31), which predicts pharmacological properties based on chemical structure, to predict G-F1 biological activity. PASS provides a list of biological activity types for which the active (Pa) and inactive (Pi) probabilities are calculated. Pa and Pi values are independent and their values vary from 0 to 1.

*Molecular docking simulation*

Molecular docking studies were performed with Autodock 4.2 (32-34) using the Lamarckian Genetic Algorithm 4.2 scoring parameter (35). The X-ray crystal structure of p38 MAPK was obtained from the RCSB PDB Database (http://www.rcsb.org/pdb) as a PDB file (PDB ID: 1A9U) with a resolution of 2.50 Å (36). Figure 2. shows the 3D structure of p38 MAPK. The enzyme structure was co-crystallized with SB203580 inhibitor in 1A9U; polar hydrogen atoms and Kollman charges were added to perform docking studies. Then, SB203580 and water molecules were removed and polar hydrogen atoms were added. SB203580 has two hydrogen bond interactions with residues Lys53 and Met109 (37). These active sites are considered to be the most favorable for docking simulations. The best molecular interaction was identified based on binding orientation of p38 MAPK key residues and their corresponding binding affinity scores. The docking results of G-F1 with p38 MAPK were visualized using the DS 3.5 program; further 2D interactions were analyzed using the PoseView web-server (38, 39) (http://poseview.zbh.uni-hamburg.de/poseview) to determine their mode of interaction with pocket residues. 

*Molecular dynamics simulations*

Molecular dynamics simulations were performed with a Gromacs96 43a2 force field (40) and a single-point-charge water model (41) using the Gromos 4.6 program (42). In this study, we performed molecular dynamics simulations for three structures, which include the apo form of p38 MAPK, the known inhibitor complex formed by p38 MAPK/SB203580, and the p38 MAPK/G-F1 complex. The Gromos program’s utility pdb2gmx was used to generate p38 MAPK topology files; topology files for ligands G-F1 and SB203580 were created using the Dundee PRODRG2 server (43) (http://davapc1.bioch.dundee.ac.uk/cgi-bin/prodrg). Each structure was neutralized by adding the appropriate ions (Na^+^ or Cl^-^), followed by canonical ensemble and isothermal-isobaric ensemble equilibration. Individual systems underwent 25-nanosecond production. The root mean square deviation (RMSD) and root mean square fluctuation (RMSF) values were calculated using the Gromos program utilities g_rms and g_rmsf. All molecular dynamics simulations were performed using the Intel® 2.93 GHz Xenon® CPU 5679 RHEL 6 server. 

## Results and Discussion

Parkinson’s disease is the most common neurodegenerative disease that effects thousands of people throughout the world. During Parkinson’s disease, there is successive neurodegeneration and loss of neurons in the brain. Recently, it has been suggested that p38MAPK plays a significant role in hippocampal neurons apoptosis and cerebellar granule neurons, (44–46). Therefore, in this study, we investigated the in silico inhibitory activity of G-F1 by assessing the expression of p38MAPK. To our knowledge this is the first report showing G-F1 has strong inhibitory action on the expression of p38MAPK and may be acts as anti-Parkinson’s disease-specific. Ginsenosides, the most active constituent of *Panax ginseng*, plays important role in the pharmacological action of ginseng. Modern studies demonstrate that these ginsenosides are distinctive triterpenoid dammarane saponins and have a valuable beneficial effect on the protection and stimulation for CNS-related diseased conditions, mainly PD. Though, these ginsenosides are being used for decades, however, their pharmacological action and molecular level activity is still unknown. P38, one of the most probable the therapeutic target of PD, and the tested ginsenoside (G-F1) for neuroprotective behavior was selected for the current study. We examined the molecular interaction using in silico molecular docking to reveal the protein-ligand (p38-G-F1) interaction. The strength of the protein-ligand (p38-G-F1) interaction were determined by the binding free energy or docking score of the protein–ligand complex structure.

*Screening pharmacological properties (ADMET properties)*

*In silico* predictions have been used to determine ADMET descriptors such as serum protein binding capacity, blood-brain barrier crossing, central nervous system activity, and HERG K+ channel activity, apparent Caco-2, apparent MDCK, and skin permeability, percentage of human gastrointestinal absorption, hepatotoxicity, and CYP2D6 probability. We used Lipinski’s rule of five to determine the drug-likeness of G-F1. By this rule, most orally administered drugs have molecular weight less than 500, a distribution ratio less than 5, less than 5 hydrogen bond donors, and less than 10 hydrogen bond acceptors. G-F1 had a molecular weight of 638.88 Kda, a distribution ratio of 2.482, 7 hydrogen bond donors and 14 hydrogen bond acceptors. Although G-F1 did not obey Lipinski’s rule of 5, these values fall within the accepted ranges of 95% of known drugs. Additionally, toxicity estimation is one of the most vital task in natural compound screening (47), thus we estimated hepatotoxicity descriptors (0 is non-toxic and 1 is toxic) for tested ginsenoside by using ADMET module accessible from DS 2.5. The detailed results of predicted ADMET values of G-F1 with acceptable range were shown in Table 1. At last, after this screening filter, we concluded that G-F1 has the capability to cross the BBB and act as potential therapeutic agents of PD treatment. Furthermore, the biological activity spectrum of G-F1 was predicted using the PASS program, which shows the probability of active (Pa) and the probability of inactive (Pi) properties. Pa and Pi range from 0 to 1. The predicted 28 properties of G-F1, along with Pa and Pi values, are shown in Table 2. 

*Molecular interaction studies*

The p38 MAPK crystal structure was used to perform docking simulation using the Auto Dock 4.2 program. In this study we used SB203580 as a control molecule, which is a well-known drug for the inactivation and inhibition of p38 MAPK via its specific active sites including Lys 53 and Met 109. Figure 4. shows the RMSD of 0.98 Å between the docked and crystal conformations of SB203580, indicating the reliability of the Auto Dock 4.2 program in reproducing the experimentally observed binding mode for p38 MAPK inhibitors. The docking results were verified based on the binding energy and formation of hydrogen bonds with Lys 53 and Met 109. The interaction between G-F1 and p38 MAPK formed four hydrogen bonds (Figure 3.) with a binding energy of -7.32 Cal/mol. The detailed docking results along with the control ligand are described in Table 3. The Lys 53 residue formed one hydrogen bond between the hydrogen and oxygen atoms of G-F1, with a hydrogen bond distance of 1.98 Å. The Met 109 residue involved two hydrogen bond interactions between the hydrogen and oxygen atoms of G-F1, with hydrogen bond lengths of 1.89 Å and 1.98 Å, respectively. The Asp 168 residue, another enzyme active site, was also involved in hydrogen bond formation (length 2.10 Å). 

*Structure stability refinement using molecular dynamics*

In order to examine and refine the structural stability of p38 MAPK, three individual molecular dynamics simulations were performed on the apo form of p38 MAPK and its ligand complexes with SB203580 and G-F1. Figure 5A. shows that the RMSD against the backbone of each complex attained equilibration around 1604 ps (p38 MAPK-SB203580), and 01438 ps (p38 MAPK-G-F1). After equilibration, each complex remained stable throughout the 25000 ps simulation time. The RMSF analysis of the p38 MAPK-G-F1 complex was performed through the entire simulation against Cα atoms. To analyze the trajectory files, the active sites of the p38 MAPK-G-F1 complex were compared with the p38 MAPK-SB203580 complex. Figure 5B. displays the RMSF values of each complex. Molecular dynamics analysis showed less fluctuation in the enzyme’s binding to G-F1 than its binding to SB203580. Throughout the simulation, the MAPK-G-F1 binding orientation did not affect structural stability or residue-level fluctuations of Cα atoms. In current research, computer docking and molecular modeling techniques play a significant role in the drug discovery and development.

*Summary*

In conclusion, the ADMET and PASS results suggest that G-F1 is a non-toxic, drug-like compound with potential for p38 MAPK inhibition. In particular, the strong interaction between G-F1 and p38 MAPK involved four hydrogen bonds with two active sites (Lys 53, Met 109). Molecular dynamics simulation suggested that the specified change less obviously was observed at coupling G-F1, p38 MAPK active site residues. Importantly, the p38 MAPK-G-F1 binding orientation did not affect the enzyme’s structural stability. Taken together, our results suggest that G-F1 has a potentially therapeutic role by inhibiting p38 MAPK. 

Our study concludes that ginsenoside F1 will be a promising compound for the development of anti-Parkinson’s therapeutic agents.
